# Comparative Study of the Tempering Behavior of Different Martensitic Steels by Means of In-Situ Diffractometry and Dilatometry

**DOI:** 10.3390/ma13225058

**Published:** 2020-11-10

**Authors:** Martin Hunkel, Juan Dong, Jeremy Epp, Daniel Kaiser, Stefan Dietrich, Volker Schulze, Ali Rajaei, Bengt Hallstedt, Christoph Broeckmann

**Affiliations:** 1 Leibniz Institut für Werkstofforientierte Technologien–IWT, Badgasteiner Straße 3, 28359 Bremen, Germany; dong@iwt-bremen.de (J.D.); epp@iwt-bremen.de (J.E.); 2 MAPEX Center for Materials and Processes, University of Bremen, Bibliothekstraße 1, 28359 Bremen, Germany; 3 Institut für angewandte Materialien-Werkstoffkunde, Karlsruher Institut für Technologie, Engelbert-Arnold-Str. 4, 76131 Karlsruhe, Germany; Daniel.Kaiser@dillinger.biz (D.K.); stefan.dietrich@kit.edu (S.D.); volker.schulze@kit.edu (V.S.); 4 Institut für Werkstoffanwendungen im Maschinenbau, RWTH Aachen University, Augustinerbach 4, 52062 Aachen, Germany; a.rajaei@iwm.rwth-aachen.de (A.R.); b.hallstedt@iwm.rwth-aachen.de (B.H.); c.broeckmann@iwm.rwth-aachen.de (C.B.)

**Keywords:** steel, tempering, dilatometry, in-situ diffractometry

## Abstract

Martensitic steels are tempered to increase the toughness of the metastable martensite, which is brittle in the as-quenched state, and to achieve a more stable microstructure. During the tempering of steels, several particular overlapping effects can arise. Classical dilatometric investigations can only detect effects by monitoring the integral length change of the sample. Additional in-situ diffractometry allowed a differentiation of the individual effects such as transformation of retained austenite and formation of cementite during tempering. Additionally, the lattice parameters of martensite and therefrom the tetragonality was analyzed. Two low-alloy steels with carbon contents of 0.4 and 1.0 wt.% and a high-alloy 5Cr-1Mo-steel with 0.4 wt.% carbon were investigated by dilatometry and in-situ diffractometry. In this paper, microstructural effects during tempering of the investigated steels are discussed by a comparative study of dilatometric and diffractometric experiments. The influence of the chemical composition on the tempering behavior is illustrated by comparing the determined effects of the three steels. The kinetics of tempering is similar for the low-alloy steels and shifted to much higher temperatures for the high-alloy steel. During tempering, the tetragonality of martensite in the steel with 1.0 wt% carbon shifts towards a low carbon behavior, as in the steels with 0.4 wt.% carbon.

## 1. Introduction

Martensite formation within steels is a widely researched phenomenon due to its industrial relevance and scientific interest. Depending mainly on the chemical composition, the properties of martensite vary in a large range. The most important alloying element controlling the mechanical properties of a steel is carbon. The hardness increases from 35 HRC for ultra-low carbon steels up to 66 HRC for Fe-0.8%C after quenching [1]. Applying a tempering after quenching, the properties of the as-quenched martensite can be balanced according to the application’s requirements. During tempering, several transformation and recovery effects occur. These tempering effects are described by the tempering stages [2,3]:

(0) Redistribution of carbon atoms (<100 °C)
(1) Formation of transition carbides (100–200 °C)
(2) Loss of tetragonality (100–200 °C)
(3) Decomposition of retained austenite (170–300 °C)
(4) Formation of cementite (250–350 °C)
(5) Recovery
(6) Formation of secondary precipitates if possible.

Studies have shown that the formation of transition carbides in stage (1) triggers the loss of tetragonality in stage (2) [4,5]. Stages (3) and (4) have a continuous overlap over a wide range [6,7,8]. It should be noted that the temperature range of the tempering effects depends strongly on the heating rate and on the chemical composition. Therefore, the given temperature ranges are considered only as a rough estimation.

Alloying elements have a strong impact on the stability of the as-quenched microstructure and the tempering behavior of the steel. Therefore, the effects during tempering vary with the chemical composition. Quenched and tempered steels like SAE 4140 have a mostly martensitic microstructure and small amounts of retained austenite. The most important effect during tempering is the precipitation of carbides [9]. In bearing steels like SAE 52100, this martensitic transformation is not complete at room temperature, resulting in a martensitic microstructure including considerable amounts of retained austenite. The retained austenite transforms during tempering accompanied by the precipitation of carbides and the loss of martensite tetragonality [10,11], and results in a different tempering behavior compared to SAE 4140. In high-alloyed hot working steels like SAE H13, the alloying elements provide enhanced wear and corrosion resistance. The microstructure of this steel group shows a secondary hardness maximum by increasing the tempering temperature, where secondary precipitates are formed [12,13]. The steels cited are all Cr- or Cr-Mo-alloyed. Depending on the Cr-Mo- and C-content, the tempering behavior differs significantly.

Microstructure evolution in steels is widely studied by dilatometry [14] or differential scanning calorimetry [2]. The advantages are a straightforward procedure and a high resolution. The main disadvantage is the integral measurement of length or heat change. Due to the integral measurement, superposed effects of carbide precipitation and loss of tetragonality cannot be separated clearly. A measurement technique to observe individual mechanisms is the time-resolved investigation of microstructure evolutions by the X-ray diffraction method [15]. Most in-situ X-ray measurements were done by using synchrotron radiation due to the much higher intensity than by conventional X-ray diffraction measurements. By in-situ synchrotron X-ray measurements, the transformation of retained austenite during tempering under stress was researched for a steel SAE 52100 [16]. It was shown that the applied stress accelerates the transformation of retained austenite. Also, by in-situ synchrotron X-ray measurements, the decrease of carbon content in martensite during tempering was determined [16,17]. The evolution of the tetragonality and the precipitates were determined for a high-alloyed martensitic steel [18]. Combined experiments using dilatometry and in-situ X-ray diffraction experiments clearly show the effects leading to a length change.

In this work, dilatometry and X-ray diffractometry were applied to investigate the tempering effects in three steel grades for a characteristic representation of different steel groups. The SAE 4140, SAE 52100, and SAE H13 steels were chosen, having the main alloying elements Cr and Mo, besides C. The tool steel SAE H13 is additionally alloyed with Si and V. These elements have a significant influence on the tempering behavior by retarding the cementite formation (Si) and vanadium carbide (VC) formation. The variation in the chemical composition leads to a significantly different tempering behavior, which is the main issue to investigate in this work. In the following, first material and methods and then the experimental results are described. Finally, the results are discussed to enlighten the similarities and differences in the tempering behavior due to the chemical composition.

## 2. Materials and Methods

### 2.1. Materials

Three different steels were investigated in this study: the low-alloyed medium-carbon steel SAE 4140 (EN 42CrMo4), the low-alloyed high-carbon steel SAE 52100 (EN 100Cr6), and the high-alloyed medium-carbon steel SAE H13 (EN X40CrMoV5-1). This choice allows the investigation of the effect of carbon as well as substitutional alloying elements on the tempering behavior. All applied steels were conventionally continuous cast and bar rolled. The respective compositions determined by emission spectral analysis are given in Table 1.

### 2.2. Heat Treatment

Typical austenitizing and quenching conditions depend on the steel composition. Consequently, the usual heat treatment conditions were applied for each of the three steels. The initial state of the SAE 4140 steel was quenched and tempered. The SAE 4140 dilatometer samples were austenitized at 920 °C for 60 s in order to comply with induction heating. Afterwards, the samples were quenched with maximum quenching rate within approximately 7 s to 30 °C. The X-ray diffraction (XRD) samples were austenitized using the same conditions in a vacuum furnace with subsequent quenching into oil. The hardness of the specimens after quenching was 730 HV0.1. The dilatometer samples were tempered with a heating rate of 5 K min^−1^ (0.083 K s^−1^) up to 600 °C.

The bearing steel SAE 52100 was delivered in a spheroidized annealed condition. This steel is typically austenitized in the two-phase field austenite-cementite. The austenitizing temperature applied was 850 °C. The austenitizing duration was 45 min to ensure a homogeneous distribution of carbon within austenite. The dilatometer specimens were quenched with 20 K s^−1^ to ambient temperature. The specimens were immediately heated up to 600 °C with a heating rate of 2 and 5 K min^−1^ (0.083 K s^−1^). The XRD samples were austenitized under the same conditions in a vacuum furnace with quenching into oil and a heating rate of 2 K min^−1^ (0.033 K s^−1^) for the subsequent tempering. It is known that the tempering behavior of SAE 52100 depends strongly on the quenching condition [10]. Therefore, the quenching was controlled as closely as possible.

The hot work tool steel SAE H13 contains the highest amounts of alloying elements among the three investigated steels. Chromium, molybdenum, and vanadium are elements with a high affinity towards carbon and carbide formation. The initial state of the SAE H13 steel was the soft annealed condition, including V- and Cr-rich carbides, which were characterized by means of energy dispersive X-ray fluorescence (EDX) analysis. Due to the high stability of the V-rich carbides, the SAE H13 is typically austenitized at higher temperatures in order to dissolve carbides in austenite. Dilatometer samples were austenitized at 1050 °C for 15 min and subsequently quenched to ambient temperature within 100 s, with a cooling rate of 6 K s^−1^ above the martensite start temperature, Ms ≅ 400 °C. A martensitic microstructure with roughly 5% retained austenite and a hardness of 650 HV5 was formed. Samples were then heated up with constant rates of 5 K min^−1^ (0.083 K s^−1^) and tempered at 650 °C for 1 h. Metallographic examinations of an as-quenched and of a quenched and tempered state are found in the Supplementary Material (Figures S3–S5).

### 2.3. Experimental Methods

#### 2.3.1. Dilatometry

The induction dilatometer TA Instruments DIL805 (New Castle, DE, USA) was used for the dilatometric measurements. The samples were cylinders of 10 mm in length and 4 mm in diameter. To ensure through-hardening, hollow samples with 3 mm inner diameter were chosen for SAE 4140. For further analyzing, the measured length change Δl was normalized with respect of the initial length $l_{0}$ of the specimen to get the relative length change, dilatation $\varepsilon =\Delta l/l_{0}$. Quenching dilatometers are inferior in precision with respect to high-precision dilatometers due to unavoidable temperature gradients within the device. The transformation kinetics were analyzed by the curves of the dilatation and the dilatation rate with respect to the temperature dε/dT during the heat treatment. The latter one shows the effects more pronounced [14]. In the curves of the dilatation rate, transformations are observed as positive or negative peaks, where the thermal expansion coefficient defines the baseline.

#### 2.3.2. In-Situ X-Ray Diffraction

For the in-situ X-ray measurements, a diffractometer (Bruker-AXS, Karlsruhe, Germany) with a built-in heating unit was used [19]. The diffractometer is equipped with a θ/2θ goniometer, a rotating anode, and a very fast position-sensitive detector (PSD). The anode material is Cobalt, and the radiation line used is Iron-filtered Kα1/2 with λ = 1.788893/1.792801 Å. The beam with a size of Ø 3 mm is supplied by a power of 34 kV and 200 mA. Measurements were performed in the 2θ-range from 43° to 103° with an increment of 0.1° and a scan speed of 0.2 s.

**Figure 1:**
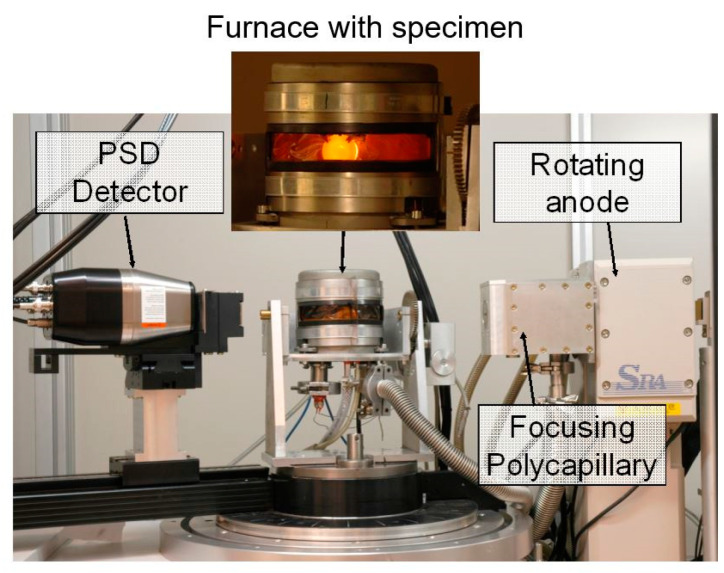
Diffractometer used for in-situ X-ray measurements with furnace (enlarged with glowing sample).

The tempering experiments are enabled in a self-built miniaturized furnace, see Figure 1, which has a heating wire, a sample holder, and a window made by Kapton in order to let the X-ray beam pass through. The samples were heated by heat conduction using resistance heating of the wire. Sample sizes are small discs with thickness of 2 mm and diameter of 22 mm (SAE 4140, SAE 52100) or diameter of 15 mm (SAE H13). The temperature is measured by a K-thermocouple inserted in the sample and controlled by a power control system. In order to protect the sample surface from oxidation, the furnace was evacuated and purged by nitrogen gas 3 times; thereafter, the tempering experiments were started. The recorded XRD diffractograms were evaluated by the Rietveld method [20]. The lattice parameters of martensite and the phase contents of martensite, austenite, and cementite were evaluated. The estimated statistic error of the determination of the phase content is about 2–3 ma%. Unfortunately, the contents of transition and alloyed carbides were too small to detect during fast in-situ measurements.

**Figure 2:**
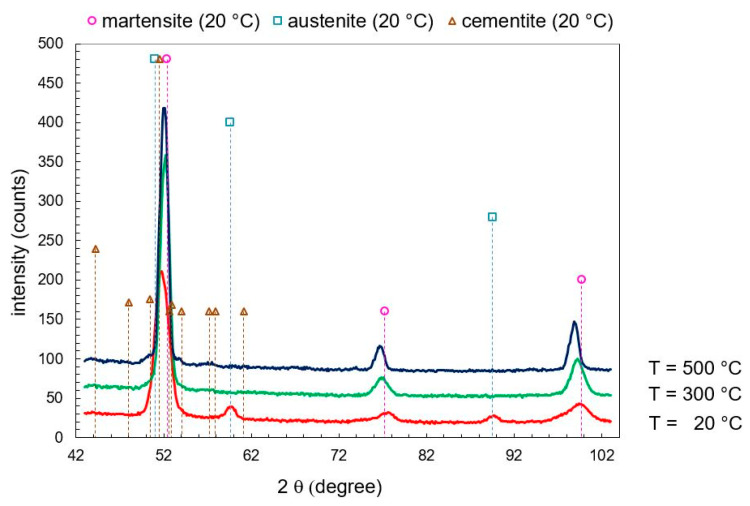
Diffraction patterns at 20, 300, and 500 °C from in-situ X-ray diffraction (XRD) for SAE 52100.

Exemplarily, the diffraction patterns for 20, 300, and 500 °C are shown for SAE 52100 in Figure 2. At 20 °C, 20 ma.% austenite, 3 ma.% cementite, and 87 ma.% martensite could be detected. The austenite peak vanished at 300 °C. At 500 °C, the cementite increased to an amount of 10 ma.%. The tetragonality resulted in a broad, asymmetric peak of martensite at 20 °C due to peak splitting. At 300 °C, the peak of martensite was more symmetric due to the loss of tetragonality. The martensite peaks were shifted to smaller angles because of thermal expansion. The diffraction patterns for SAE 4140 and SAE H13 determined at 20, 300, and 500 °C are found in the Supplementary Material (Figures S1 and S2).

## 3. Results and Interpretation

### 3.1. Comparison of Dilatometer Results

**Figure 3:**
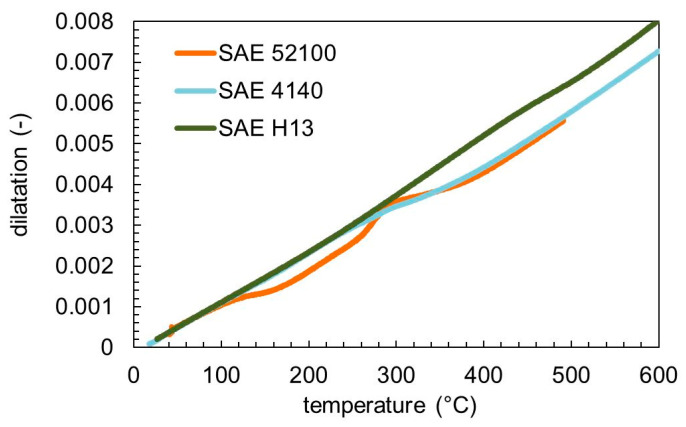
Measured dilatation for the three used steels tempered with a heating rate of 5 K min^−1^.

**Figure 4:**
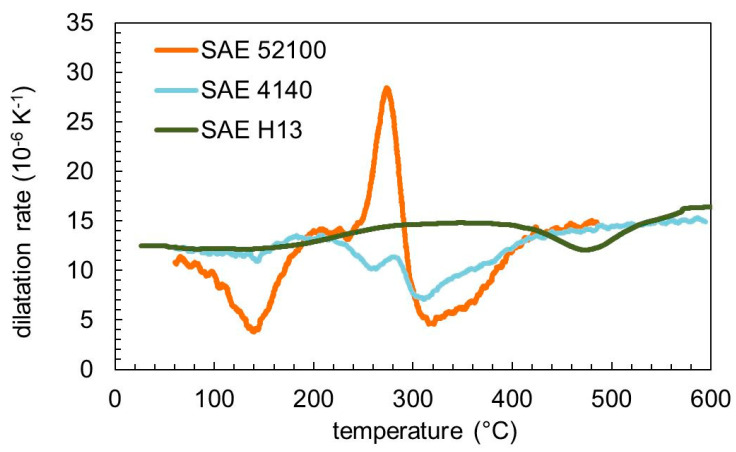
Dilatation rate for the three used steels tempered with a heating rate of 5 K min^−1^.

The tempering behavior of the three steels was compared using the same heating rate of 5 K min^–1^. In Figure 3, the measured dilatation is plotted, and the resulting dilatation rate is presented in Figure 4. The thermal expansion coefficient is about 14 × 10^−6^ K^−1^ in the first stage of tempering. Tempering effects can be detected as a peak-like deviation from the thermal expansion coefficient in Figure 4. The differences in the tempering behavior for the three steels can be clearly seen. The thermal expansion coefficient of the final martensite is 14.5 × 10^−6^ K^−1^ for SAE 4140 and SAE 52100 and 16.5 × 10^−6^ K^−1^ for SAE H13.

### 3.2. Dilatometry and Phase Content of SAE 4140

**Figure 5:**
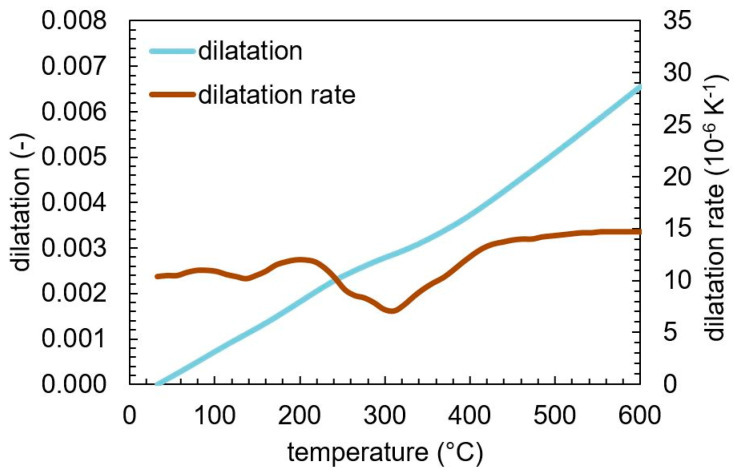
Dilatation and dilatation rate as a function of temperature for SAE 4140 during tempering with a heating rate of 5 K min^−1^.

The resulting dilatometric curve with the derivative with respect to temperature is shown in Figure 5. Between approximately 80–200 °C, a dip in the dilatation derivative can be found. Usually, tempering stage 1, the precipitation of ε-carbides, is found to occur in this temperature regime [2,3,21], whereas a recent paper [22] claims that this dip is due to a carbon redistribution during tempering. From 240 to 400 °C, a second dip is visible, which is much more pronounced compared to the first one. In this regime, the decomposition of retained austenite as well as the formation of cementite is known to occur [2,8]. The plateau in the dip that can be observed at approximately 260–300 °C might indicate the decomposition of retained austenite, which results in a dilatation increase, compared to the carbide precipitation, which results in a decrease. After reaching 400 °C, solely an increase in the thermal expansion coefficient is found, indicating no further changes with increasing temperature.

**Figure 6:**
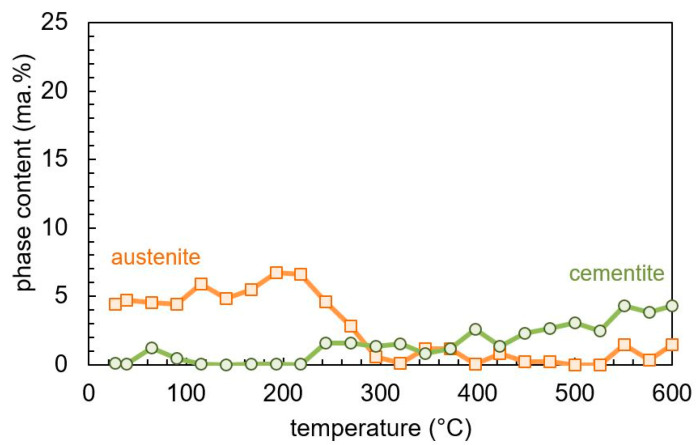
Phase content evolution during tempering of martensitic SAE 4140 determined by in-situ X-ray measurement.

Figure 6 shows the evolution of the phase contents of martensite, retained austenite, and cementite during tempering, as determined by in-situ X-ray analysis. A retained austenite content of approximately 5 ma.% was found at the beginning, which decomposes between 270 and 300 °C. At the same time, the content of cementite starts to increase. This substantiates the assumption that the plateau observed in the derivative of the tempering dilatation between 270 and 300 °C is caused by the decomposition of retained austenite into ferrite and cementite.

With further increasing tempering temperature, a continuous increase in the cementite content can be observed. This is contradictory to the dilatometry and literature results [2,8], which indicate the cementite precipitation to be finished by reaching about 400 °C. However, one should keep in mind that the amount of cementite is at the detection threshold of the X-ray measurements, which introduces large scatter in the quantitative values.

### 3.3. Dilatometry and Phase Content of SAE 52100

**Figure 7:**
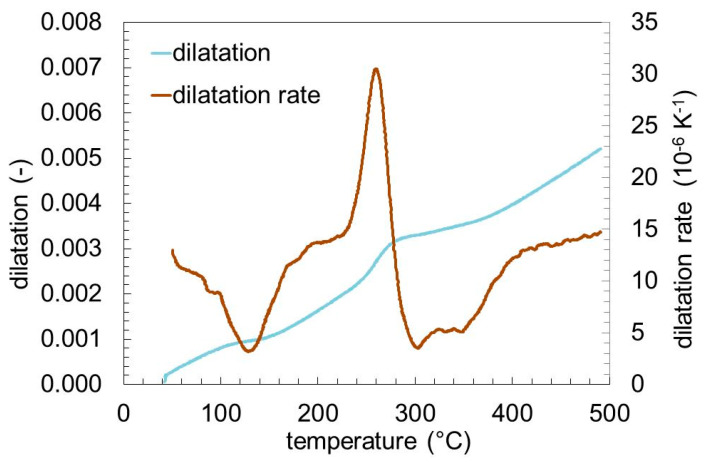
Dilatation and dilatation rate of SAE 52100 during tempering with a heating rate of 2 K min^−1^.

Figure 7 shows the dilatation and dilatation rate during tempering. The baseline of dilatation rate is the thermal expansion coefficient of about 14·10^−6^ K^−1^. There is the evidence of 4 events given by the deviation from this baseline. The negative peak at 125 °C and the positive peak at 260 °C are clearly visible. There is a broad negative peak around 300 °C. Additionally, there is a small negative peak at about 360 °C.

**Figure 8:**
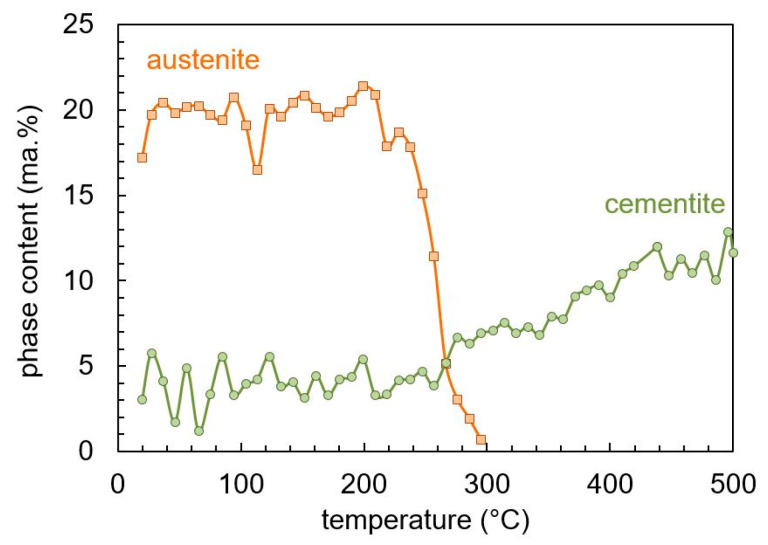
Phase contents of SAE 52100 during tempering determined by in-situ X-ray measurement.

Figure 8 shows the phase contents of retained austenite, cementite, and martensite. The retained austenite transforms in the temperature range between 200 and 300 °C corresponding to the positive peak at 260 °C in Figure 7. The initial cementite content of about 4 ma.% corresponds to the amount of undissolved carbides after hardening, and is consistent to previous investigations [19]. The cementite formation during tempering starts at the same temperature, which the retained austenite starts to transform. The formation of cementite is much slower than the transformation of the retained austenite, leading to a continuous increase of cementite over the entire temperature range. Corresponding to the decrease of austenite and the increase of cementite, the martensite/ferrite content changes.

### 3.4. Dilatometry and Phase Content of SAE H13

**Figure 9:**
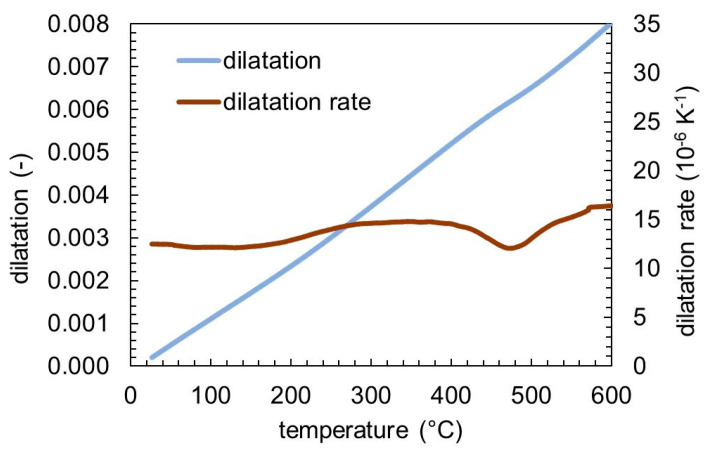
Dilatation and dilatation rate of SAE H13 during tempering with a heating rate of 5 K min^−1^.

Figure 9 shows the dilatation and the dilatation rate of the SAE H13 sample, measured by dilatometry. Similar to the other two steel grades, two stages of slight volume change can be observed that are attributed to the *ε*-carbide precipitation (100–200 °C) and cementite formation (380–500 °C).

**Figure 10:**
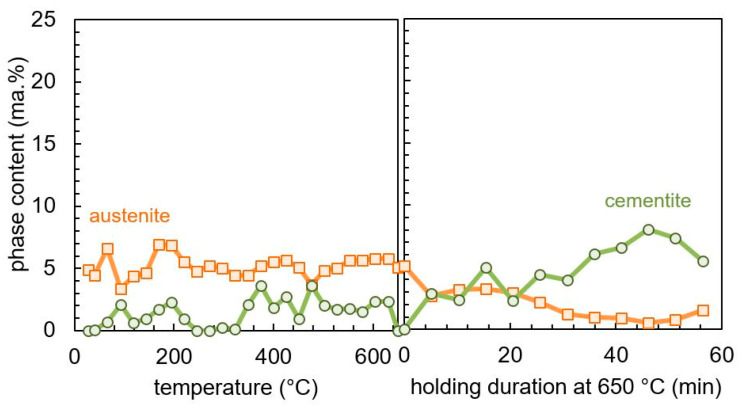
Measured microstructure evolution during tempering of SAE H13 during heating to 650 °C with 5 K min^−1^ (**left**) and holding at 650 °C (**right**), determined by in-situ X-ray measurement.

The evolution of the content of retained austenite and cementite during heating and 1 h of tempering at 650 °C is shown in Figure 10. The retained austenite stays stable up to 650 °C and then transforms over the holding time. Cementite content shows only a small positive trend during heating up, which corresponds qualitatively to the second peak of the dilatation rate shown in Figure 9. However, the content of cementite is apparently less than the scattering of the measured data, so that a quantitative conclusion cannot be drawn. During the tempering at 650 °C, more cementite is formed, and a clear rise of the phase content can be observed. The precipitation of fine V-, Mo-, and Cr-rich carbides during the tempering of the high-alloy steel SAE H13 is another major difference, compared to the tempering behavior of SAE 4140 and SAE 52100 [12]. The investigation of these nano-sized carbides requires a highly resolved measurement technique such as transmission electron microscopy (TEM) and cannot be observed by means of dilatometry and X-ray diffraction. Thus, the formation of these carbides is not discussed in this work.

### 3.5. Lattice Parameters and Tetragonality of Martensite

**Figure 11:**
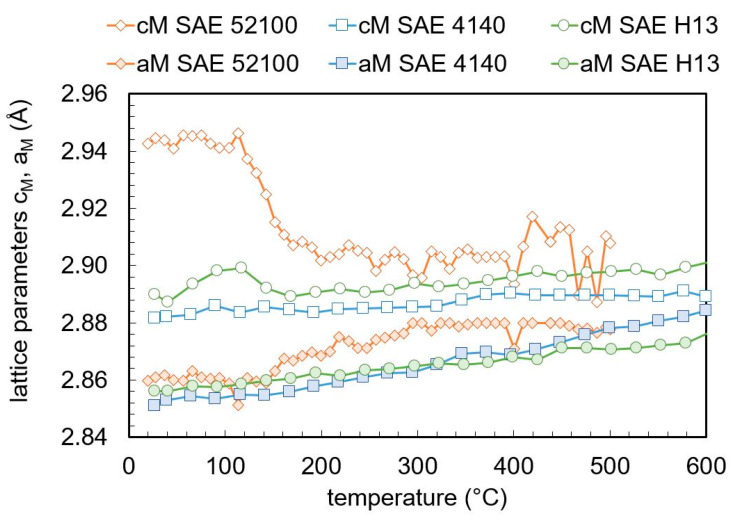
Evolution of martensite lattice parameters during tempering determined by in-situ X-ray measurement.

The lattice parameters a_M_ and c_M_ for martensite are plotted in Figure 11 for the three steels. The lattice parameters for SAE 4140 and SAE H13 increase linearly with temperature. Between 100 and 200 °C, for SAE 52100, the lattice parameter c_M_ drops significantly and the lattice parameter a_M_ increases nonlinearly.

**Figure 12:**
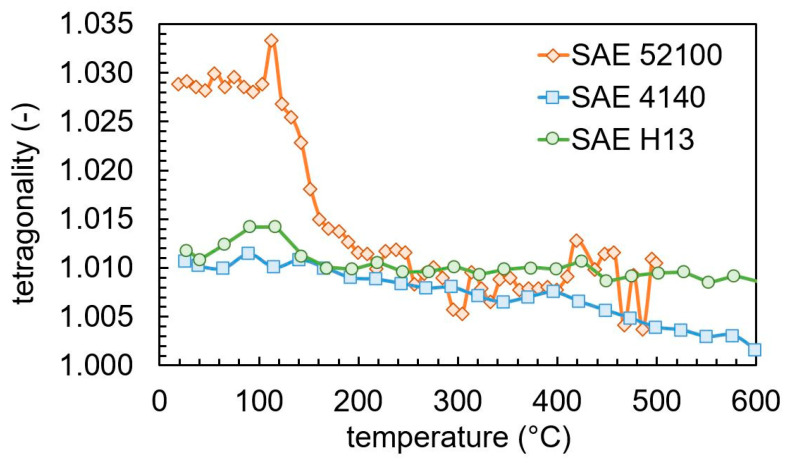
Evolution of martensite tetragonality during tempering.

The ratio between the lattice parameters a_M_ and c_M_ of martensite, which describes the tetragonality, is given in Figure 12. For SAE 52100, the tetragonality drops sharply between 100 and 200 °C. For both SAE 4140 and SAE H13, the tetragonality decreases linearly with the temperature. The decrease is larger for SAE 4140. Despite the significantly larger carbon content of SAE 52100, the tetragonality is nearly the same in the temperature region of 200–400 °C as for SAE 4140 and SAE H13.

## 4. Discussion

### 4.1. Phase Transformations and Precipitation

For all three steel types, the decomposition of retained austenite could be clearly observed. When comparing SAE 4140 and SAE 52100, the in-situ XRD measurements reveal that the decomposition temperature range of both steels lies between approximately 250 and 300 °C. The major difference is given by the initial content of retained austenite of 5% and 20% respectively, due to the significantly lower martensite start temperature resulting from the higher carbon content in SAE 52100. In both cases, this range coincides with a positive peak in the dilatation rate. This is expected since the austenite phase has a close packed fcc lattice. However, the authors of Reference [23] stated that depending on the carbon content of the retained austenite, a contraction can occur during decomposition of retained austenite due to a less densely packed fcc lattice from the carbon content dissolved in the lattice. This could not be observed for the SAE 4140 and SAE 52100 steels investigated here, which differ significantly in carbon content. This temperature range was also found in Reference [2,24] for a steel with similar composition as SAE 52100. For the medium carbon steel SAE 4150 (EN 50CrMo4), the authors of Reference [22] found a higher temperature range between approximately 350 and 400 °C for the decomposition of retained austenite, which could not be supported for the very similar SAE 4140 in this study. The hot-working tool steel SAE H13 contains a similar carbon content as the SAE 4140, which results in the same initial amount of retained austenite of approximately 5%. In contrast to the other two steels, the decomposition of retained austenite only occurs during the isothermal hold at 650 °C. For each of the three steels, the transformation of retained austenite and the cementite formation overlap as reported before [6,7,8]. Surprisingly, this is the also case for the high-alloy steel with significantly higher transformation temperature. Possibly, cementite formation relaxes internal stresses due to the smaller martensite volume. Resulting the relaxing stresses triggers the transformation of the retained austenite, which is bainite-like. The transformation of the retained austenite is therefore shifted to higher temperature, as Si retards the cementite precipitation.

The cementite precipitation of the steels SAE 4140 and SAE 52100 was found from the XRD measurements starting around 250 °C and was not finished at the final temperature. Cementite volume contents observed are close to the quantification limit of the measuring method applied with high time resolution. Cementite precipitation is observed during the dilatometry measurements as a negative peak in the dilatation rate curve. This minimum does not have the expected form of the derivative of a sigmoid function, which is explained by the effect of the simultaneous decomposition of retained austenite at the early stage of the cementite precipitation regime, as discussed above. For the high-alloy steel SAE H13, the cementite precipitation is shifted to the holding step at 650 °C, similar to the transformation of the retained austenite. It is assumed that the higher Si content in the high-alloy steel SAE H13 prevents cementite precipitation and stabilizes the retained austenite [25,26]. Independent of the transformation temperature, for all steels, the cementite formation and the retained austenite transformation overlaps, as already reported [6,7,8].

The evolution of dε/dT of SAE H13 with increasing temperature shows two pronounced minima. The first one lies in the same temperature range as for the two other steels, the magnitude of the minimum is comparable to the one of SAE 4140 and smaller than for SAE 52100. This minimum can again be attributed to the precipitation of ε-carbides. The second minimum lies around 470 °C and coincides with the rise of the cementite fraction as measured by XRD, although a precise determination of the beginning of the cementite precipitation is difficult due to its small amount. In contrast to the other steels, the minimum of dε/dT has the shape of a single peak as it can be expected for a single-phase transformation, since the counteracting decomposition of retained austenite occurs at a higher temperature.

### 4.2. Lattice Parameter and Tetragonality

**Figure 13:**
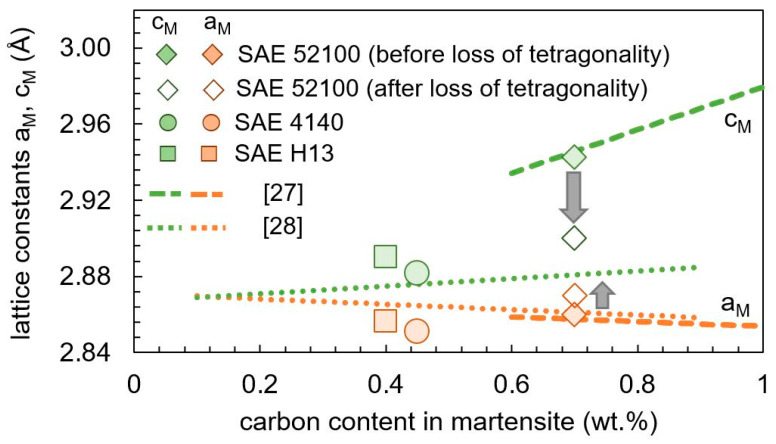
Experimental findings and literature reference [27,28] of lattice parameters a_M_ and c_M_ as function of the carbon content at room temperature. For SAE 52100, the lattice parameters before and after tempering stage (2) are plotted.

Above 0.6 wt.% carbon, the lattice parameters a_M_ and c_M_ depend linearly on the carbon content, independent of further alloying elements [27,28]. Below 0.6 wt.% carbon, the lattice parameters can deviate from the linear behavior, regardless of the carbon content and depends on the steel composition [28,29]. Figure 13 compares the lattice parameters with the high carbon reference [27] and a low carbon reference [28]. It has to be kept in mind that only the carbon in solid solution influences the martensitic lattice parameters, while the carbon content bound in the carbides has no influence on it. For SAE 4140 and SAE H13, a negligible amount of carbides is assumed after quenching and the carbon content is given by the nominal carbon content in Table 1. For SAE 52100, approximately 4% cementite was detected in the as-quenched state. As a result, the solute carbon content in martensite is approximately 0.7 wt.% [15]. The lattice constants of SAE 52100 before tempering stage (2), loss of tetragonality, fits the high carbon reference very well. Tempering stage (2) was not detected for SAE 4140 and H13. After the sharp change in the lattice parameters for SAE 52100 between 100 and 200 °C in Figure 12, the determined lattice parameters fit better to a low carbon behavior [28]. Both SAE 4140 and SAE H13 fit the low carbon reference better.

The tetragonality of the as-quenched martensite of SAE 4140 and SAE H13 is nearly equal to 1.01, while it has a significantly higher value of 1.03 in SAE 52100, due to the higher carbon content. Varying the carbon content in solution, the tetragonality of as-quenched martensite is between 1.005 and 1.015 for a carbon content below 0.5 wt.% and above 1.025 for a carbon content above 0.5 wt.% [29,30]. While a rapid decrease in tetragonality is found during the tempering of SAE 52100 from 1.03 to approximately 1.01 between 100 and 200 °C, only a small change is found in this temperature range for SAE H13 and SAE 4140.

**Figure 14:**
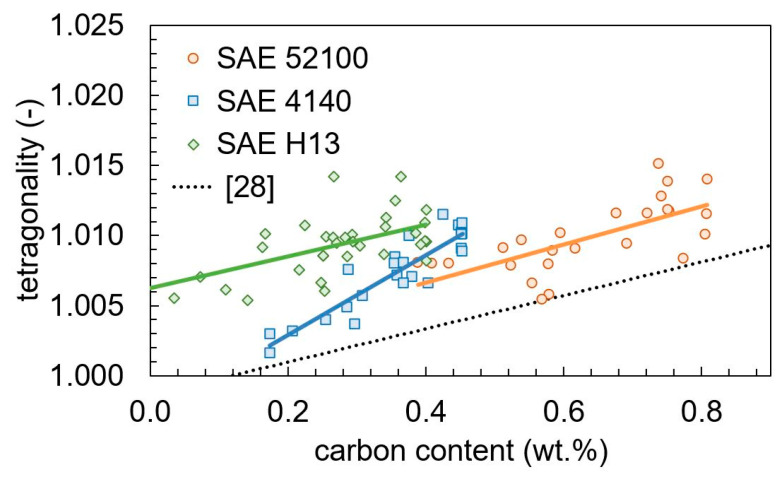
Tetragonality in dependency on the calculated carbon content compared with a literature reference [28].

The tetragonality decreases significantly in the two medium carbon steels only above approximately 200 °C, the temperature range where cementite formation was also found. The carbon content within martensite $c_{M}$ was calculated by a lever rule:

(1) $$c_{M}=\frac{c_{0}-c_{C}p_{C}}{p_{M}},$$

using measured martensite and cementite contents $p_{M}$ and $p_{C}$, mean carbon content $c_{0}$, and carbon content of cementite $c_{C}$. The tetragonality depends linearly on the carbon content with different slopes below and above 0.6 wt.% carbon [27,30,31]. Cementite formation leads to a decrease of dissolved carbon. The carbon content within martensite was estimated from the cementite content. In Figure 14, the tetragonality is plotted against the calculated carbon content. For SAE 52100, the measurements below 200 °C are not plotted. For all three steels, a linear dependency of the tetragonality on the carbon content is given. The slope depends on the steel composition. As found before, the tetragonality does not only depend on the carbon content [29].

### 4.3. Dilatometry

Comparing the dilatometer results with the in-situ X-ray measurements, the individual effects can be identified more clearly. Detected tempering effects are:

⮚ A pronounced loss of tetragonality with negative peak occurs at 150 °C for SAE 52100 (tempering stage (2)). Tempering stage (2) does not appear for SAE 4140 and SAE H13.
⮚ Retained austenite transforms with a positive peak at 170 °C for SAE 52100 and 180 °C for SAE 4140 (tempering stage (3)). The peak is more pronounced for SAE 52100 due to the higher retained austenite content. For SAE H13, retained austenite transforms during holding at 650 °C.
⮚ Formation of cementite and loss of tetragonality with a broad negative peak occurs between 200 and 430 °C for SAE 52100 and SAE 4140 and between 490 and 550 °C for SAE H13 (tempering stage (4)).

Due to the linear correlation between cementite content and low carbon tetragonality, as shown in Figure 13, a distinction between cementite precipitation and loss of tetragonality cannot be done by dilatometry.

Transition carbides could not be detected by in-situ X-ray diffraction due to the fast measurements leading to low counting statistics. Nonetheless, a further peak in the dilatometer results was found, which could be interpreted by the literature [5,9,10]:

⮚ Formation of transition carbides leads to a negative peak between 50 and 200 °C for SAE 52100 and SAE 4140 (tempering stage (1)). The transition carbide peak for SAE H13 is between 50 and 300 °C.

The thermal expansion coefficient of the final martensite depends only weakly on the chemical composition.

### 4.4. Influence of Chemical Composition on Tempering Behavior

In this work, two low-alloy steels with carbon contents of 0.4 and 1.0 wt.% and a high-alloy steel with 0.4 wt.% carbon were investigated. The retained austenite content of the three steels depends mainly on the carbon content, and the retained austenite is transformed during tempering. Also, the amount of cementite depends on the carbon content. While for both low-alloy steels these transformations are in the same temperature region, they are shifted to a higher temperature range for the high-alloy steels. Significantly higher contents of Mo, Cr, or Si stabilize the martensite against cementite formation. It was already demonstrated that cementite precipitation and retained austenite transformation overlap over a wide range of temperature independently [8]. Regardless of the steel composition, this was also found in this work. The as-quenched state of the SAE 52100 has a significantly higher tetragonality than SAE 4140 and SAE H13 due to the higher carbon content. The tetragonality of SAE 52100 after quenching fits the literature reference for carbon contents higher than 0.6 wt.% [27]. After the loss of the high carbon tetragonality during tempering stage (2), SAE 52100 shows a low carbon tetragonality, as SAE 4140 and SAE H13, with a weak dependency on the dissolved carbon content in martensite. The loss of tetragonality, retained austenite content, and cementite formation results in large effects in the dilatometer curve for SAE 52100, leads to smaller effects for SAE 4140, and only to very small effects for SAE H13.

## 5. Conclusions

Three different steels, SAE 4140, SAE 52100, and SAE H13, were investigated by dilatometry and corresponding in-situ X-ray diffraction to determine similarities and differences during tempering. The steel compositions were chosen in a way that different effects could be identified clearly. Effects analyzed were lattice parameters, martensite tetragonality, retained austenite transformation, and cementite precipitation. The main effects determined from peaks in the dilatation rate can be identified clearly by in-situ X-ray-diffraction. For the low-alloyed steels SAE 4140 and SAE 52100, these effects were found in a similar temperature range, while they were shifted to much higher temperatures for the high-alloy steel SAE H13. Nonetheless, retained austenite transformation and cementite precipitation are in the same temperature range for each steel. The lattice parameters of martensite show a typical evolution for low carbon steel (*c* < 0.5%). This is also valid for SAE 52100 after the loss of tetragonality during tempering. The tetragonality depends linearly on the carbon content of martensite with different slopes for each steel.

## Figures and Tables

**Table 1:** Mean chemical composition determined for the three applied steels (in wt.%).

Steel	C	Cr	Mo	Mn	Si	S	V	Ni	Cu	N
SAE 4140	0.45	0.99	0.16	0.71	0.21	0.02	-	-	-	<0.005
SAE 52100	0.97	1.50	0.10	0.43	0.21	0.004	-	0.24	0.1	<0.005
SAE H13	0.40	5.37	1.34	0.30	0.97	-	1.22	-	-	<0.005

## Supplementary Materials

The following are available online at https://www.mdpi.com/1996-1944/13/22/5058/s1, Figure S1: Diffraction patterns at 20 °C, 300 °C, and 500 °C from in-situ X-ray diffraction for SAE 4140, Figure S2: Diffraction patterns at 20 °C, 300 °C, and 500 °C from in-situ X-ray diffraction for SAE H13, Figure S3: Micrographs of SAE 4140: (a) as quenched, (b) quenched and tempered up to 600 °C, Figure S4: Micrographs of SAE 52100: (a) as quenched, (b) quenched and tempered up to 500 °C, Figure S5: SEM micrographs of SAE H13: (a) as quenched, (b) quenched and tempered up to 650 °C.
