# Supplementary material for: Comparative Study of the Tempering Behavior of Different Martensitic Steels by Means of In-Situ Diffractometry and Dilatometry
Source: Materials (Basel). 2020 Nov 10;13(22):5058. doi: 10.3390/ma13225058 (PMC7696766; doi:10.3390/ma13225058)
Supplement: Supplementary file 1 [file materials-13-05058-s001.pdf]

materials

Supplementary Materials

Comparative Study of the Tempering Behavior of Different Martensitic Steels by Means of In-Situ Diffractometry and Dilatometry

Martin Hunkel [1,*], Juan Dong [1], Jeremy Epp [1,2], Daniel Kaiser [3], Stefan Dietrich [3], Volker Schulze [3], Ali Rajaei [4], Bengt Hallstedt [4] and Christoph Broeckmann [4]

[1] Leibniz Institut für Werkstofforientierte Technologien–IWT, Badgasteiner Straße 3, 28359 Bremen, Germany; dong@iwt-bremen.de (J.D.); epp@iwt-bremen.de (J.E.)
[2] MAPEX Center for Materials and Processes, University of Bremen, Bibliothekstraße 1, 28359 Bremen, Germany
[3] Institut für angewandte Materialien - Werkstoffkunde, Karlsruher Institut für Technologie, Engelbert-Arnold-Str. 4, 76131 Karlsruhe, Germany; Daniel.Kaiser@dillinger.biz (D.K.); stefan.dietrich@kit.edu (S.D.); volker.schulze@kit.edu (V.S.)
[4] Institut für Werkstoffanwendungen im Maschinenbau, RWTH Aachen University, Augustinerbach 4, 52062 Aachen, Germany; a.rajaei@iwm.rwth-aachen.de (A.R.); b.hallstedt@iwm.rwth-aachen.de (B.H.); c.broeckmann@iwm.rwth-aachen.de (C.B.)
* Correspondence: hunkel@iwt-bremen.de

1. Diffraction Patterns

1.1. The Diffraction Patterns for SAE 52100 Is Shown in the Main Text

Exemplarily, the diffraction patterns for 20 °C, 300 °C, and 500 °C are shown in Figure S1. At 20 °C mainly martensite and a small amount of 5 ma.% austenite could be detected. The austenite peak vanished at 300 °C. At 500 °C, a peak of about 3 ma.% cementite occurred. Thermal expansion shifted the martensite peaks to smaller angles. To further elucidate the effects from the dilatometric measurements, they are compared to the results from the in-situ XRD tempering experiments, where lattice parameters of martensite, its tetragonality and the phase contents of cementite, martensite and retained austenite were evaluated.

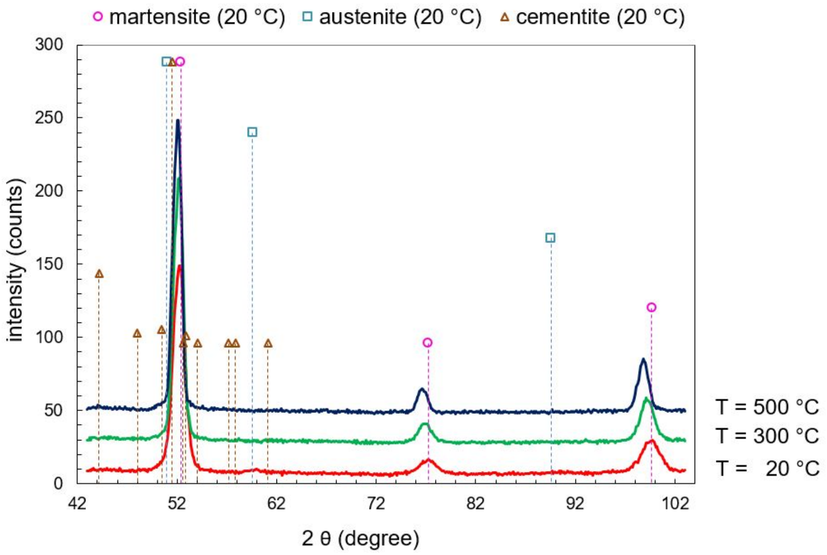

Figure S1. Diffraction patterns at 20 °C, 300 °C, and 500 °C from in-situ X-ray diffraction for SAE 4140.

The diffraction patterns for 20 °C, 300 °C, and 500 °C are shown in Figure S2. At each temperature, mainly martensite and a small amount of 5 ma.% austenite could be detected. Thermal expansion shifted the martensite peaks smaller angles.

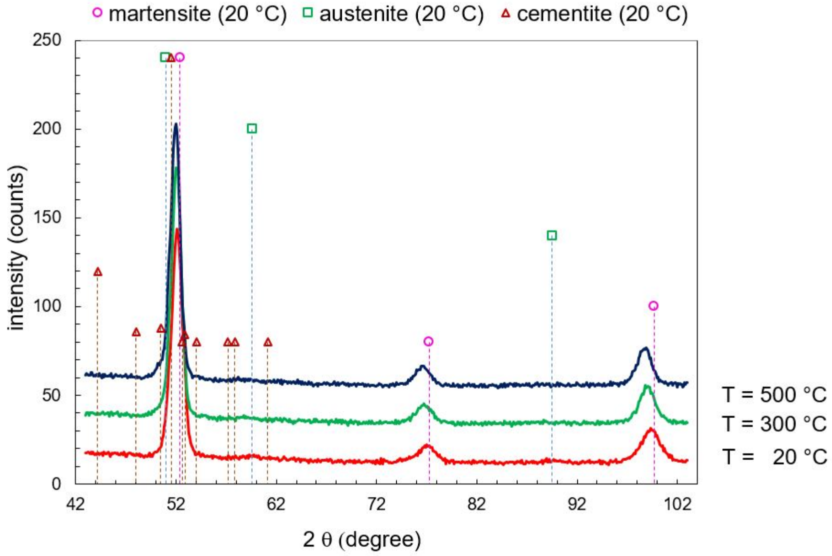

Figure S2. Diffraction patterns at 20 °C, 300 °C, and 500 °C from in-situ X-ray diffraction for SAE H13.

1.2. Metallography

The microstructure of as-quenched SAE 4140 consists of full martensite, see Figure S3a. Partially, some martensite laths can be identified. Figure S3b shows micrographs of the quenched and tempered microstructure.

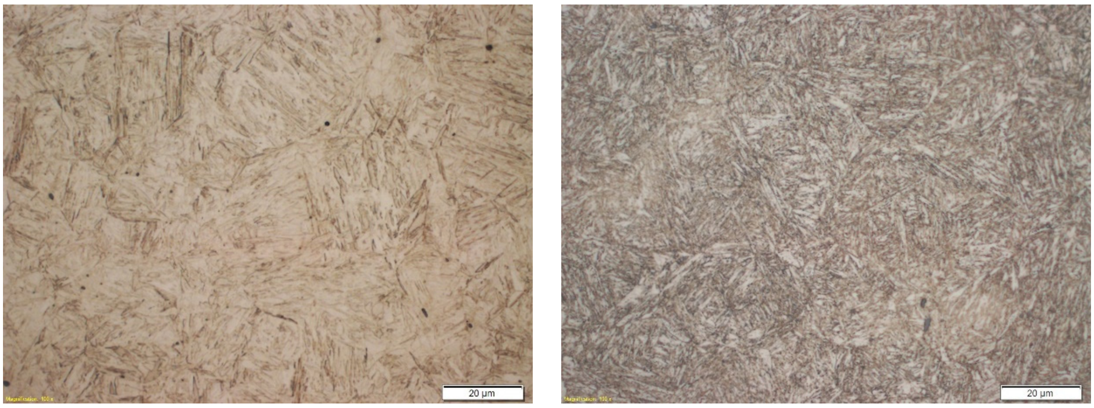

a) **b**)

Figure S3. Micrographs of SAE 4140: **a**) as quenched, **b**) quenched and tempered up to 600 °C.

Figure S4a shows the micrograph of as quenched SAE 52100 (etchant: Nital). For the austenitizing condition applied, a significant amount of cementite is undissolved. This undissolved cementite can be identified by white-etched, spherical precipitations. Further, brown etched martensite plates and brighter austenite can be identified. Quenched and tempered SAE 52100 is shown in Figure S4b. The microstructure consists of a typical high annealed microstructure of SAE 52100.

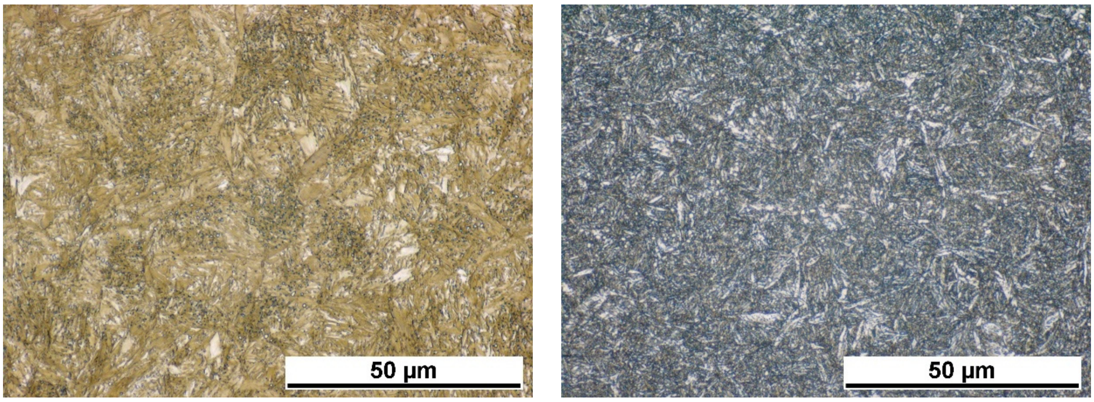

a) **b**)

Figure S4. Micrographs of SAE 52100: **a**) as quenched, **b**) quenched and tempered up to 500 °C.

An SEM micrograph of as-quenched SAE H13 is shown in Figure S5a. The microstructure is a typical martensite with some undissolved, spherical carbides. Figure S5b shows the quenched and tempered state. The secondary electron images (SE) were acquired by using a Helios Nanolab DualBeam microscope of type G3 CX with an accelerating voltage of 5 kV and a beam current of 0.17 nA.

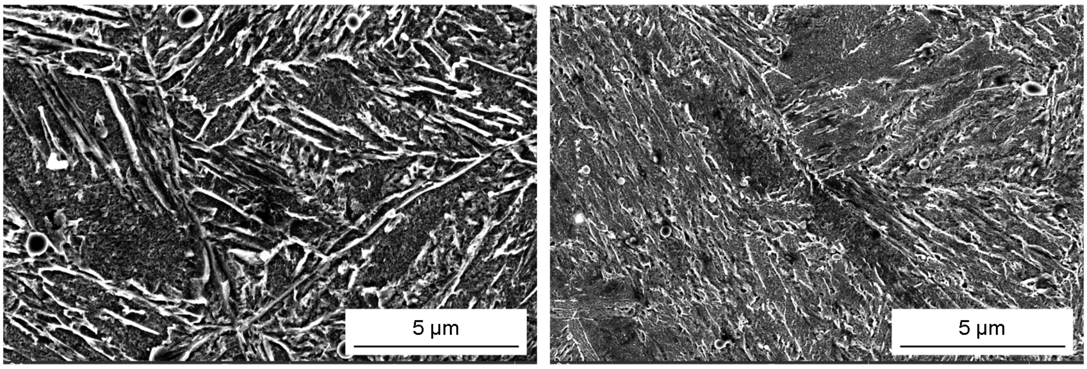

a) **b**)

Figure S5. SEM micrographs of SAE H13: **a**) as quenched, **b**) quenched and tempered up to 650 °C.

© 2020 by the authors. Submitted for possible open access publication under the terms and conditions of the Creative Commons Attribution (CC BY) license (http://creativecommons.org/licenses/by/4.0/).
